# Task Switching: On the Relation of Cognitive Flexibility with Cognitive Capacity

**DOI:** 10.3390/jintelligence11040068

**Published:** 2023-03-30

**Authors:** Florian Schmitz, Raimund J. Krämer

**Affiliations:** Department of Psychology, University of Duisburg-Essen, Universitätsstraße 2, 45141 Essen, Germany

**Keywords:** task switching, cognitive flexibility, cognitive capacity, visuospatial working memory, diffusion model, latent difference modeling

## Abstract

The task-switching paradigm is deemed a measure of cognitive flexibility. Previous research has demonstrated that individual differences in task-switch costs are moderately inversely related to cognitive ability. However, current theories emphasize multiple component processes of task switching, such as task-set preparation and task-set inertia. The relations of task-switching processes with cognitive ability were investigated in the current study. Participants completed a task-switching paradigm with geometric forms and a visuospatial working memory capacity (WMC) task. The task-switch effect was decomposed with the diffusion model. Effects of task-switching and response congruency were estimated as latent differences using structural equation modeling. Their magnitudes and relations with visuospatial WMC were investigated. Effects in the means of parameter estimates replicated previous findings, namely increased non-decision time in task-switch trials. Further, task switches and response incongruency had independent effects on drift rates, reflecting their differential effects on task readiness. Findings obtained with the figural tasks employed in this study revealed that WMC was inversely related to the task-switch effect in non-decision time. Relations with drift rates were inconsistent. Finally, WMC was moderately inversely related to response caution. These findings suggest that more able participants either needed less time for task-set preparation or that they invested less time for task-set preparation.

## 1. Introduction: Intelligence as Capacity and Flexibility

### 1.1. Overview

Competing theories postulate alternative mechanisms and processes as a basis of psychometric intelligence. Among the most prominent are cognitive capacity and cognitive flexibility, and both may overlap conceptually or depend on each other. We seek to contribute to this special issue on “Cognitive Flexibility” by offering a closer inspection of one of the most prominent laboratory paradigms used to assess cognitive flexibility, namely task switching, its component processes, and their respective relations with cognitive capacity.

First, we will briefly argue why we need explanatory approaches in intelligence research. Then, we will offer a structured overview of relevant findings in task-switching research. This will cover a brief introduction into commonly employed experimental paradigms, the most prominent theoretical accounts, and the relations of observed task-switching costs with measures of cognitive capacity. Further, we will address diffusion modeling of response times that has been suggested to decompose relevant component processes of task switching. Results obtained in previous studies using these process decomposition techniques will be briefly considered.

Most of these previous findings concern group differences. The aim of the present study is to test if individual differences in these component processes can be reliably modeled using latent difference scores. Further, their relations with individual differences in cognitive capacity were tested. In line with the notion that these basic processes may constitute the fluid basis of intelligence, we chose to employ visuospatial paradigms with geometric forms that can be argued to be largely free of prior knowledge.

### 1.2. Searching the Basis of Intelligence

Intelligence has been argued to be both a success story and a failure: success because of its predictive utility, but a failure because it lacks a satisfactory explanation (Birney and Beckmann 2022; De Boeck et al. 2020). Theories of intelligence have traditionally relied on factor-analytic work. This approach has stimulated the development of highly reliable and predictively valid tests. Ironically, the success of traditional test development may have impeded thorough efforts to research the cognitive basis of intelligence. The absence of a satisfactory theory may, in turn, contribute to acceptance problems of intelligence tests in spite of their high psychometric qualities (Sparfeldt et al. 2022). Consequently, more research has been advocated to understand the cognitive basis of intelligence (Birney and Beckmann 2022; Conway et al. 2021; Frischkorn et al. 2022; Goecke et al. 2021; Van Der Maas et al. 2017). Previous research has linked fluid intelligence with higher-order cognitive functions such as working memory capacity (e.g., Wilhelm et al. 2013) and cognitive flexibility (e.g., Schneider and McGrew 2012). In turn, capacity and flexibility have been suggested to rely on lower-level cognitive functions such as attention, shifting, inhibition, and relational binding (Birney and Beckmann 2022). Both perspectives, their relations, and possibly shared constituent processes will be briefly considered below.

Consider first cognitive capacity. Accounting for fluid intelligence in terms of working memory capacity (WMC) has become very popular since both constructs have been shown to be substantially related (Buehner et al. 2005; Oberauer et al. 2007; Süß et al. 2002; Wilhelm et al. 2013). This account appears straightforward as virtually all higher cognitive functions require the temporary storage and manipulation of information, which are the defining characteristics of working memory.

In turn, a number of lower-level functions and processes have been proposed as determinants of WMC. A prominent account of WMC is the binding hypothesis that explains cognitive capacity in terms of building, maintaining, and updating relations in working memory (Oberauer et al. 2007; Wilhelm et al. 2013). This account on relational integration has been supported by demonstrating differential relations of binding capacity with classical indicators of WMC (e.g., complex span tasks) and tests of fluid intelligence (Bateman et al. 2017; Chuderski 2014). Further accounts of WMC that have received much attention include mental speed (Barrouillet et al. 2007, 2011), executive attention (Engle 2002; Kane et al. 2004), secondary memory (Unsworth and Engle 2007; Unsworth et al. 2009), and general discrimination ability (Troche and Rammsayer 2009; Troche et al. 2014). All these low-level mechanisms have been proposed to contribute partially to the capacity of working memory and thereby ultimately to explaining fluid intelligence. The Process Overlap Theory (Kovacs and Conway 2016) offers a comprehensive framework that seeks to integrate all partial accounts and posits that the positive manifold observed in ability measures results from their shared basal cognitive functions and processes (Kovacs and Conway 2019, see also Thomson 1916).

Naturally, all these low-level functions as assessed by means of elementary cognitive tasks (ECTs) will only offer a partial explanation of WMC and ultimately of intelligence. However, it is well known from mental speed paradigms that their predictive strength expands as their complexity and difficulty increases (Deary 2001; Sheppard and Vernon 2008). Complexity refers to the nature and number of component process required for the completion of a task (Beckmann et al. 2017; Birney and Beckmann 2022) and should not be equated with psychometric difficulty, although both can be expected to be related. Confirming the complexity hypothesis, it has been shown that performance in more complex ECTs that supposedly require more component processes are better predictors of WMC and fluid intelligence (Schmitz and Wilhelm 2016; Schmitz et al. 2018). Further, using bifactor modeling it was shown that the specific variance associated with more complex tasks was incrementally predictive of intelligence over and above a general factor of mental speed modeled across all tasks (Lerche et al. 2020). While results of the latter studies could as well reflect confounding variables between different tasks, the effect has been confirmed when experimentally manipulating complexity within one task. Specifically, it has been demonstrated that increasing the number of bindings required within one ECT increases its relation with WMC (Goecke et al. 2021).

Consider next cognitive flexibility. Some theories emphasize that fluid intelligence is best viewed as cognitive flexibility (Birney and Beckmann 2022; Schneider and McGrew 2012). This implies that intelligence can be best detected in novel situations for which there are no overlearned schemata, hence, where people need to adapt flexibly to the requirements of the given circumstances. The account on cognitive flexibility may help explain individual differences in complex real-world situations. However, this theory has received less attention in mainstream intelligence research, and this may be partly because the assessment of cognitive flexibility is not trivial. Relevant paradigms suggested for its assessment include creativity tasks (that typically intend to measure facets like fluency, flexibility, and originality; Carroll 1993), complex problem–solving scenarios (Beckmann et al. 2017; Dörner and Funke 2017), experiential learning tasks such as cards-selection paradigms (e.g., the IGT, Bechara et al. 1994, or the WCST, Berg 1948), and task-switching paradigms (Allport et al. 1994; Meiran 1996; Rogers and Monsell 1995).

All these paradigms have certain challenges from a psychometric perspective. Creativity tasks can be difficult to score objectively (Weiss et al. 2021). Complex scenarios and experiential learning potentially measure the interaction of several traits or component processes (Gansler et al. 2011). In contrast, task switching paradigms are relatively well understood in terms of their processing requirements (Koch et al. 2018), and they can be scored objectively and reliably. However, they conceptualize flexibility narrowly in terms of switching between pre-defined task-sets. Since rule inference and inferential learning are not required, task switching paradigms do not grasp the entire complexity of flexible adjustment (Yu et al. 2019).

Naturally, the accounts on cognitive capacity and cognitive flexibility are not mutually exclusive and rather complement each other. First, cognitive capacity may be argued to be the basis also for coping effectively with novel situations. Capacity is relevant for maintaining, combining, and processing interim results. Not surprisingly, WMC has been shown to predict success in experiential learning tasks (Schmitz et al. 2020) and in complex scenarios (Stadler et al. 2015). Second, both cognitive capacity and flexibility may share certain basal cognitive process and functions. Adaptivity in complex and novel situations has been argued to require rapid and flexible encoding, representation of information, and manipulation of relations between aspects of the physical and mental world (Beckmann 2014). Thus, its basal cognitive mechanisms comprise mental speed, updating of bindings, and task switching.

### 1.3. The Task Switching Paradigm

Compared to other experimental paradigms suggested for the assessment of cognitive flexibility, task switching is relatively well-defined in terms of its requirements. Additionally, it has received much attention in experimental cognitive psychology and cognitive neuroscience (Kiesel et al. 2010; Koch et al. 2018).

In experimental research, a task set comprises which attribute of the stimulus to attend to and how these are mapped to response values (Monsell et al. 2000). In essence, the core of a task-set can be characterized as a set of S-R bindings (e.g., in a color classification task with binary responses: red → left; blue → right). Task sets allow coherence of action across time, even in face of interference. At the same time, switching between task sets should require flexible shifts in behavior when external or internal demands change (Mayr 2001).

There has been a long tradition of task-switching paradigms in psychological research, and methods to assess cognitive flexibility have been continuously refined. In early task-switching research, for instance, using the plus–minus task (Jersild 1927), overall RT differences between mixed-task and task-pure blocks have been interpreted as global task switch costs. Conversely, the differences between task-switch trials and task-repeat trials within the same mixed blocks have been used as an index of local task-switch costs. This removes some of the confounding factors resulting from overall block differences, such as learning and motivation. However, if tasks are presented in a predictable sequence (e.g., AABB, as in the classical number-letter task, Rogers and Monsell 1995), participants may complete some or most of the task-switch preparatory processes before the stimulus appears, at least when the response–stimulus interval is sufficiently long.

This can be prevented when using the explicit-cueing paradigm, where task-switches occur unpredictably within one block. The relevant task in each trial is indicated by an explicit task cue. Variation of the cue-stimulus interval (while keeping the response–stimulus interval constant) has been used to study the effects of task preparatory processes (Meiran 1996). Naturally, task-switch costs are the largest when the task cue is presented simultaneously with the imperative stimulus. One such example of an explicit cueing paradigm with simultaneous cue-stimulus onset is the color-size switching task with geometric forms (Klauer et al. 2010). In this task, geometric forms are either classified according to their color or to their size, and the task-cue is a stimulus attribute, namely whether the shape is framed or filled. The color-size switching task has further advantages, for instance, that it does not require participants to be capable of basal arithmetic operations and not even to be familiar with numbers and letters.

### 1.4. Theories of Task Switching

Alternative accounts have been proposed to explain the task-switching effect (Koch et al. 2018). These comprise theories that emphasize task-set preparation, inertia from a previous task set, and multiple components accounts.

The classical account on task-set reconfiguration (Rogers and Monsell 1995) assumes that switching costs arise from (additional) preparatory processes in task-switch trials compared with task–repeat trials that have to be completed before the stimulus can be processed. Increased error rates in task-switch trials are suggested to result from incomplete task-set preparation in these trials. Accounts that postulate task-switch specific preparatory processes are referred to as additional process accounts (De Jong 2000; Rogers and Monsell 1995; Rubinstein et al. 2001). Theories of task-set preparation include the re-programming of the cognitive system (Ruthruff et al. 2001), top-down biasing of the relevant task-demand units (Gilbert and Shallice 2002), retrieving the new task set from long-term memory and loading it into working memory (Goschke 2000; Mayr and Kliegl 2000), or loading it into the region of direct access of procedural working memory (Risse and Oberauer 2010). These theories are supported by the finding that task-switch costs are considerably reduced when a valid task-cue is presented well before the stimulus (Meiran 1996). A sufficiently long cue-stimulus interval would allow complete task-set preparation largely prior to stimulus onset.

By contrast, the account on task-set inertia (Allport et al. 1994; Allport and Wylie 2000) explains switching cost by a more passive mechanism, namely persisting activation of the task set of the previous trial. Such inertia effects would be beneficial in task-repeat trials but adverse in task-switch trials. Both forms of pro-active interference would contribute to the observed difference in task performance between trial types. In support of the task-set inertia account, it has been shown that switching effects are not confined to the trial directly following a task switch. They decrease consistently across trials since the competing task set has been executed the last time (Monsell 2003). Further evidence exploits the so-called backward inhibition effect (Koch et al. 2010; Mayr and Keele 2000), according to which a previous task set is inhibited when it interferes with response selection in the current task set. This inhibition also persists, which makes it more difficult to switch back to the inhibited task set. The n-2 repetition cost describes the finding that participants find it harder to switch to a task set that they have recently switched away from (e.g., in an ABA task sequence) as compared with a task set that has not been recently executed (e.g., CBA) (Koch et al. 2010; Mayr and Keele 2000). Further, people find it harder to switch to a dominant task set as compared to switching to a less dominant task (Allport et al. 1994; Allport and Wylie 2000). This noteworthy effect has been explained analogously by dominant task sets requiring stronger inhibition, thus, strong inhibition will also persist. Interestingly, the backward-inhibition effect was shown not to depend on the cue-stimulus interval, but rather on the experience of response conflict (Mayr and Keele 2000; Schuch and Koch 2003). This suggests that inertia effects may be generally more automatic in terms of persisting activation or stimulus-triggered re-activation.

In fact, it has been proposed that task-switching effects in the explicit-cueing paradigm can be explained in the absence of executive control, namely by means of compound-cue retrieval of the correct response (Logan and Bundesen 2003; Schneider and Logan 2005). Specifically, it was suggested that task cue and stimulus are encoded and form a compound cue that determines the correct response. In case of task-repeat trials, cue encoding would be faster, as a trace is still found in short-term memory. This would explain the observed differences in response time between task-repeat and task-switch trials. However, some findings are difficult to reconcile with a compound cueing account alone. In case of task-switching paradigms with two cues per task, there are still n-2 repetition costs even when the cue is exchanged (Altmann 2007; Gade and Koch 2008). This suggests that inhibition takes place on the level of task sets, and thus gives evidence of their existence. Another interesting finding is that task-switching costs have been observed only when task rules have been explicitly instructed but not when all individual S-R mappings were instructed (Dreisbach et al. 2006, 2007). This look-up table effect is arguably difficult to explain on the basis of compound cueing. However, the parsimony of Logan’s account is elegant. And it should be kept in mind that the task-switch effect may be partially attributed to repetition benefits in task-repeat trials.

To summarize, task switching costs have been explained by two lines of theorizing: An explanation in terms of task-set preparation emphasizes additional processes in task-switch trials that may be exerted as a form of proactive control (De Jong 2000; Rogers and Monsell 1995). Task-set inertia has been proposed as a more automatic effect, and its direction depends on whether a task-repeat or a task-switch trial is executed (Allport et al. 1994; Allport and Wylie 2000). However, reactive control may be triggered even at a late stage when response conflict is perceived (Schuch and Koch 2003). Given that there is convincing evidence for both mechanisms, most theorists today favor multiple-component models of task switching (Mayr and Kliegl 2003; Ruthruff et al. 2001). These seek to integrate all relevant mechanisms into a joint process model. Preparation is usually conceived to take place in an earlier phase, whereas inertia effects would occur in a later phase when the stimulus is processed. Naturally, proactive and reactive mechanisms are not independent. The more complete the task-set preparation, the more efficient should be stimulus processing in relative terms.

### 1.5. Relation of Task Switching with WMC

Task shifting has been suggested to be an executive function along with inhibition and updating (Miyake et al. 2000). This classical three-partite taxonomy assumes all functions to be at least moderately related, with task switching appearing to draw on both other functions. Task-set preparation corresponds with an updating of relevant S-R bindings (i.e., proactive control), and inhibition applies when interference is experienced during response selection (Schuch and Koch 2003) (i.e., reactive control). Further, updating appears to overlap with binding processes both conceptually and empirically (Oberauer et al. 2007; Wilhelm et al. 2013). While interference control has been argued to be a general basis of all control functions (Miyake and Friedman 2012), the question arises how and to what extent task shifting is related with updating or binding capacity, respectively. Given that higher WMC facilitates maintaining more task-sets in an activated state, shifting should be facilitated. Similarly, it can be expected that more efficient updating of bindings should speed-up task-set preparation.

Several studies have confirmed a moderate relation between shifting and working memory capacity. Typically, task-shifting has been scored in these studies in terms of (inverse) local task-switch costs in response times, whereas WMC has been indexed as the overall proportion of correct responses in binding/updating or complex-span tasks. When both shifting and WMC have been modeled as factors with different experimental paradigms as indicators, the latent relation has been typically in the magnitude of ca. ρ ≈ .30 (Feng et al. 2022, ρ = .22, Himi et al. 2019, ρ = .33, Klauer et al. 2010, Study 1: ρ = .31; Study 2: ρ = −.11, Markett 2013, ρ = .26, Miyake et al. 2000, ρ = .56, Oberauer et al. 2003, ρ = .29). Other research using only one task per factor but several task blocks as observed indicators have found a negligible relation (Benedek et al. 2014, ρ = .01), which points to considerable task specificity.

Analogous results have been found in studies that report observed relations between single task indicators, although much weaker on average and more variable across indicators (Hambrick and Altmann 2015, r = −.07, Krumm et al. 2009, −.04 ≤ r ≤ .37, Oberauer et al. 2003, .08 ≤ r ≤ .15). Further, some research has identified moderators of the relationship: Stronger relations between shifting and WMC have been found when the switching score comprises an error penalty rather than considering only RTs (Draheim et al. 2016; Knapp 2019). This finding could be reconciled with adverse effects of differential speed-accuracy trade-offs. There is evidence also that WMC and shifting are more strongly related when the time available for set-preparation in the task-switching paradigm is short as compared to when it is long (Butler et al. 2011). This may indicate that WMC is relevant primarily when there is a requirement to finish updating quickly.

### 1.6. The Diffusion Model for Response Time Data

The diffusion model for response time data (Ratcliff 1978; Ratcliff and Rouder 1998; Voss et al. 2004) decomposes a binary decision process into a set of psychologically meaningful parameters. It is assumed that information is continuously accumulated in the form of a stochastic diffusion process that is driven by systematic stimulus-based information and by random noise. Once a response threshold is reached, the according response is elicited. Specifically, the diffusion model decomposes the decision process into a non-decision time and the actual decision process. The non-decision time (*t*_0_) has been conventionally interpreted as capturing basal stimulus encoding before the decision process and motor execution time following the decision process. The main parameters of the decision process are the drift rate (υ) and the response–caution parameter (*a*). Drift rate corresponds with the mean slope of the evidence accumulation process and has been interpreted as an indicator of processing efficiency. Conversely, the response–caution parameter corresponds with the distance of the response thresholds of the decision process and determines the speed–accuracy trade-off. The full diffusion model comprises more parameters such as a bias in starting point and inter-trial variabilities of the mentioned parameters. However, they are less frequently used for the modeling of individual differences.

The diffusion model has become increasingly popular in experimental research as it uses exhaustively the information in the response time distributions of correct and erroneous responses, and it provides a set of theoretically informative parameters (Voss et al. 2013) that can be used to delineate effects of theoretical interest that are not clearly reflected in conventional scores such as mean response times (White and Curl 2018).

Individual differences researchers have started to use the diffusion model (Kyllonen and Zu 2016; Schubert et al. 2016), particularly for performance in ECTs. These studies have confirmed that drift rates in ECTs are moderately related with ability (Ratcliff et al. 2010; Schmiedek et al. 2007; Schulz-Zhecheva et al. 2016). Further, drift rates in more complex ECTs have been found to be more highly related with WMC (Goecke et al. 2021; Schmitz et al. 2018) and intelligence (Lerche et al. 2020; Schmitz and Wilhelm 2016) as compared to simpler ECTs.

Concerning response caution, some research suggests that more able participants may exercise lower caution in simple ECTs (Schmitz and Wilhelm 2016; Schmitz et al. 2018). This appears to correspond with the finding that more able persons tend to have more favorable ability self-concepts (see Freund and Kasten 2012, for a meta–analysis). However, other research did not find cognitive ability to be related with the setting of the speed-accuracy trade-off in working memory tasks (Endres et al. 2015, using a linear ballistic accumulator model). Arguably, the setting of response caution may depend on task requirements, and some research has demonstrated differential domain-specificity of response caution (Meyer et al. 2019). Conversely, the speed-accuracy settings may be adjusted flexibly in line with the perceived difficulty of the particular task. Adaptive adjustment of caution has been demonstrated also using time-on-task analyses of tests of fluid intelligence (Becker et al. 2016; Goldhammer et al. 2015).

Finally, the non-decision time parameter has generally received less attention in research. This may be due to that its interpretation as largely reflecting motor-execution time is less of interest from a cognitive process perspective.

### 1.7. Diffusion Modeling of the Task-Switching Effect

Diffusion modeling has been suggested as a way to statistically dissociate some of the processes comprised in multiple-component models of task switching (Mayr and Kliegl 2003; Ruthruff et al. 2001). Specifically, it has been proposed (Karayanidis et al. 2009, 2010; Schmitz and Voss 2012, 2014) that the additional time required for the preparation of a new task set (De Jong 2000; Rogers and Monsell 1995) is reflected in prolonged non-decision time in task-switch trials relative to task-repeat trials. The increase in non-decision time may, thus, index proactive control processes. Conversely, effects of task-set inertia (Allport et al. 1994; Allport and Wylie 2000), both in terms of persisting activation and inhibition (Schuch and Koch 2003) would be reflected in the drift rates of the stimulus processing phase. It should be noted, though, that drift rates are influenced by both task sequence (i.e., repeat vs. switch) and task predictability (i.e., advance task cues vs. simultaneous task cues). Thus, it is likely that drift-rates are affected by task-set inertia, but also proactive task-set preparation and finally reactive interference-control at response selection (Karayanidis et al. 2009; Schmitz and Voss 2012). Effects in drift rates may therefore best be described as reflecting the overall state of task readiness. Diffusion modeling of task switching data has confirmed that task-switching costs can be reflected in all three main parameters: non-decision time, drift rates, and response caution.

Longer non-decision times have been observed in switch trials as compared to task-repeat trials in several studies whenever the new task set cannot be prepared in advance, either because it cannot be predicted with certainty or because there is not sufficient preparation time (Boag et al. 2021; Ging-Jehli and Ratcliff 2020; Karayanidis et al. 2009, 2010; Klauer et al. 2007; Madden et al. 2009; Miller et al. 2018; Schmitz and Voss 2012). Further, the increase in *t*_0_ has been shown (Schmitz and Voss 2014) to be indeed related to task-switch processes and is not confined to cue-retrieval processes, as postulated by the compound cueing account (Logan and Bundesen 2003; Schneider and Logan 2005). Non-decision time increases in task-switch trials can be low or absent in the explicit cueing paradigm (e.g., Cohen Hoffing et al. 2018) when the new task set cued sufficiently long in advance to allow for task-set preparation prior to stimulus onset. Likewise, the increase can be absent in a switching paradigm with voluntary task switches where the entire response–stimulus interval can be used for task-set preparation (Imburgio and Orr 2021).

Lower drift rates in task-switch trials relative to task-repeat trials have been shown in many studies (Boag et al. 2021; Karayanidis et al. 2009, 2010; Madden et al. 2009; Schmitz and Voss 2012, 2014; Velichkovsky et al. 2020). However, substantial task-switch effects in drift rates have not been confirmed in all studies (Miller et al. 2018). This inconsistency may reflect that drift rates could be affected by multiple factors, in particular, passive inertia but also controlled task-set preparation. In fact, some evidence suggests that the magnitude of effects in drift-rate depends on specifics of the employed switching paradigm as well as characteristics of the sample such as age of the participants (Ging-Jehli and Ratcliff 2020).

Finally, some studies report increased response caution in switch trials relative to task repeat trials when participants know the nature of the next task early enough to adjust their level of response caution (Boag et al. 2021; Ging-Jehli and Ratcliff 2020; Karayanidis et al. 2009; Mansfield et al. 2011; Schmitz and Voss 2012; Velichkovsky et al. 2020). This suggests that participants may be able to adjust their level of response caution in a trial-by-trial manner, taking into consideration how error-prone the next trial is. However, these trial-wise adjustments in response caution have not been found in all studies (Miller et al. 2018; Schmitz and Voss 2014; Weeda et al. 2014), and some evidence suggests that the trial-wise adjustment observed in young adults vanishes with age (Karayanidis et al. 2011). In yet other studies, response caution has been constrained to be equal across switch and repeat trials (Madden et al. 2009; Ong et al. 2019), which appears to be a plausible assumption when the nature of the next task cannot be predicted in advance (Karayanidis et al. 2009; Schmitz and Voss 2012).

### 1.8. Correlates of Diffusion Model Parameters

Some research has addressed correlates of diffusion model parameters estimated from task-switching paradigms, showing their utility for specific research questions. In particular, there is much developmental research revealing that component processes may be differentially linked with age.

For instance, a study with youths of different age groups (7–21 years) suggests that task-switch effects in drift rate and in non-decision time follow different trajectories. The authors (Weeda et al. 2014) interpret this as evidence that maturation of inertia-related switch costs takes place earlier than that associated with task-set preparation. Data collected from elderly people confirmed their well-known higher levels of response caution. Further, it revealed both higher non-decision time as well as higher drift-rate switching costs in elderly people (Ging-Jehli and Ratcliff 2020; Whitson et al. 2012). Another study compared three groups, namely younger adults and two elderly groups, one without and one with mild cognitive impairments (Velichkovsky et al. 2020). Both elderly groups revealed larger task-switch costs in drift-rate as compared with the younger group, but only the elderly group with cognitive impairments revealed larger switch-costs also in the non-decision time.

Generally, these findings suggest that non-decision time may be a sensitive marker of cognitive performance: This could be reconciled with its later maturation and its decline in the elderly with cognitive impairments. However, proactive control processes reflected in non-decision time could reflect both, more time required because of cognitive deficits or a compensatory strategy to reduce effects of interference. Such changes in processing mode could be conscious (strategic preparation to reduce risk of errors) or not (driven by experienced reduction of error rates). The latter can be reconciled also with the finding that people with increased state anxiety have more pronounced non-decision time switching costs (Hartanto and Yang 2022). Again, it could either indicate that anxious participants had less available resources to efficiently prepare the upcoming task or that they chose a more cautious response mode.

Other research has used modeling of the component processes in task switching for diverse research questions. For instance it has been shown that training effects in task-switching were largely driven by lowering the response caution with practice (Cohen Hoffing et al. 2018). In another line of research, the diffusion model has been used to inspect specific inhibition process within task-switching paradigms. This confirmed persisting inhibition of both response and task set in younger and older adults (Schuch 2016; Schuch and Konrad 2017), at least under conditions of episodic interference (Kowalczyk and Grange 2020). Interestingly, some research using the diffusion model suggests that n-1 task-switch costs are differently composed than n-2 repetition costs (Hartmann et al. 2019). However, no studies have elucidated how the component processes of task switching, as indicated by the diffusion model parameters, are related with cognitive ability.

### 1.9. Psychometric Challenges

Simulation studies offer evidence that diffusion model parameters can be estimated reliably for the use of individual differences research, at least when sufficient trials are available (Lerche et al. 2017; Ratcliff and Childers 2015). This was demonstrated when parameters are estimated for a single condition. Further, parameters estimated from real participant data reveal to be temporarily stable across time (Lerche and Voss 2017; Miller et al. 2018; Schubert et al. 2016), in particular drift rates. This suggests they possess trait-like characteristics.

The situation looks different, though, when difference scores are computed between two conditions, as has been traditionally done when estimating effects of executive control (e.g., Miyake et al. 2000; Stahl et al. 2014). In fact, there is evidence that difference scores computed from diffusion model parameters are particularly spurious (Grange and Schuch 2022) which impedes testing their relations with correlates of interest. The lack of reliability stems from at least two sources. First, there is noise in the parameter estimation process (Schmiedek et al. 2007), making them problematic for the study of individual differences. Second, difference scores per se are notoriously unreliable, and their reliability decreases as the correlation of their constituent scores increases (Guilford 1954; Raykov 1999). This is particularly detrimental for RT-based scores, as individual differences in the conditions that constitute the score tend to be substantially related. These high correlations are also typically observed for diffusion model parameters such as drift rates, even when modeled from different tasks (Lerche et al. 2020; Schmiedek et al. 2007).

Such reliability issues may be less of a problem in experimental research, where sufficient data is available from all participants to derive reliable mean estimates for the experimental conditions. However, it can turn out to be a challenge when individual differences are concerned (Grange and Schuch 2022; Schmiedek et al. 2007). One possible solution is to treat parameter estimates as error-prone indicators of latent parameter factors that can be modeled by means of structural equation modeling. This has been done successfully in a couple of studies with estimates derived from a single task condition (Schmiedek et al. 2007; Schmitz and Wilhelm 2016; Schulz-Zhecheva et al. 2016), and analogously, difference scores could be modeled as latent differences (McArdle and Hamagami 2001; McArdle 2009).

### 1.10. Aim of this Contribution

This contribution seeks to decompose component processes of task switching by fitting the diffusion model as suggested in previous research (e.g., Karayanidis et al. 2009, 2010; Schmitz and Voss 2012, 2014). Going beyond a replication of mean effects on diffusion model parameters, this study aims at testing their differential relations with WMC as a basis for any higher cognitive functioning. As we were interested in the fluid basis of intelligence, we decided to use figural stimuli for both the switching task and the WMC paradigm. Another advantage of using paradigms with overlapping stimulus content is that they can be expected to share overlapping working memory resources.

**Hypothesis** **1** **(H1:** **effects** **in** **t_0_).** *People higher in WMC consume less time for task-set preparation. In line with the binding account on WMC (Oberauer et al. 2007; Wilhelm et al. 2013), we predicted that people who are generally more efficient in updating bindings are also faster in updating the S-R bindings that constitute the task set. Further, the larger WMC, the more complete could task sets be maintained in memory. This prediction could be derived also from the look-up table effect (Dreisbach et al. 2006, 2007) which postulates that people do not reveal task-switch costs at all when they are capable of maintaining the entire set of relevant S-R mappings in memory.*

**Hypothesis** **2** **(H2:** **effects** **in** **ν).** *People higher in WMC are more efficient in stimulus processing, in particular, in the more complex conditions, that is (H2a) in task-switch trials and (H2b) in trials with response–incongruent stimuli. This hypotheses was derived from previous research showing that drift rates in elementary cognitive tasks are generally related with WMC (Schmiedek et al. 2007), but that this relation increases as the complexity of the elementary cognitive task increases (Goecke et al. 2021; Schmitz et al. 2018). More efficient processing in the context of interference would also be predicted from the WMC account on executive attention (Engle 2002; Kane et al. 2004).*

**Hypothesis** **3** **(H3:** **effect** **in** **a).** *People higher in WMC act less cautiously when the task at hand is relatively simplistic. This secondary hypothesis was derived from few previous observations suggesting that more able participants might exercise less response caution in elementary cognitive tasks (Schmitz and Wilhelm 2016; Schmitz et al. 2018; but see Endres et al. 2015).*

## 2. Materials and Methods

### 2.1. Sample and Procedure

A total of *N* = 101 research participants (88 female; 13 male) completed the study. Their mean age was 21.5 years (*SD* = 2.9; range 18–32), and most of them reported to be university students (*n* = 97). Data collection took part in form of group testing with up to six participants in the room.

All tasks were administered in a computerized fashion on 14-inch notebooks. The battery comprised of a task-switching paradigm, a binding/updating WMC task, and few demographic questions. All tasks were controlled by compiled C++ programs using SDL libraries for stimulus presentation and response collection. The entire battery of tasks took about 40 min to complete. Participants received partial course credit or 8 Euros as compensation. The study complied with ethical guidelines of the department, and written informed consent had been collected from all research volunteers prior to participation.

### 2.2. Task-Switching Paradigm

**Figure 1:**
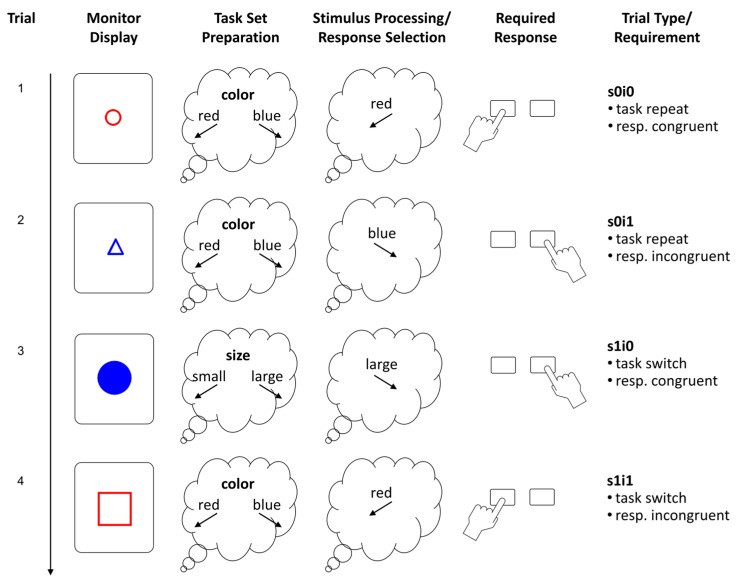
Schematic of the task-switching paradigm. The leftmost column shows the monitor display, the next columns illustrate the task-set preparation and stimulus processing requirements as well as the required response. The rightmost column presents the trial type as defined by task sequence and response–congruency of the task-relevant and irrelevant stimulus attributes (i.e., size vs. color). The trial code specifies the processing requirements, whether the trial necessitates a task switch (s1) or not (s0), and whether the response–relevant and irrelevant stimulus attributes are associated with incongruent responses (i1) or not (i0). Trial 1 would be a task-repeat trial if the preceding warm-up trial (not shown here) was a color classification trial, too. Trial types were balanced but their pseudo-random sequence was unpredictable for participants.

We employed a color-size switching task with geometric shapes (see Figure 1) that had been used in previous research (e.g., Klauer et al. 2010; Stahl et al. 2014). Geometric shapes varied in several attributes, namely shape (triangles, squares, and circles), size (small vs. large), color (red vs. blue), and whether they were filled with color or just framed. The first task was to classify shapes according to their size, where small forms and large forms had to be classified by pressing a left and right key, respectively. The second task was to classify shapes by their color, where red and blue forms had to be sorted to the left and right, respectively. The task cue was whether the shape was filled or framed, where filled shapes had to be classified according to size and the framed shapes according to color. The shape varied but was not relevant for the task. There were two task-pure blocks of 44 trials each (first size then color classification) and a mixed practice block with 24 trials. Then, followed the three (mixed) test blocks of 128 trials (plus eight warm-up trials) each. Pseudo-random trial lists were used to standardize the procedure for the assessment of individual differences. Task sequence (repeat vs. switch), response compatibility of the stimulus attributes, and the type of shape were balanced.

Each trial started with the presentation of a shape in the center of the monitor that remained visible until the correct response had been given. The left and right control keys on the notebook keyboard served as left and right response buttons. In case of a classification error, a dark gray “X” was displayed beneath the stimulus and the correct response was required in order to strengthen the mapping rules, and for enforcing that the intended task set was executed on that trial. Then, the screen was cleared and remained blank for a response–stimulus interval of 500 ms. (Technically, the cue–stimulus interval was 0 ms, as the cue was a stimulus attribute.) Small and large shapes were about two and four cm in diameter, respectively, and primary colors were used for the red (RGB = 255, 0, 0) and the blue shapes (RGB = 0, 0, 255). The background was light gray (RGB = 220, 220, 220). Mapping reminders were presented on the left and right side of the monitor and remained visible thought the entire block. Trials from the three mixed blocks (excluding the short training block and the warm-up trials) were used for the analyses. Since latency and accuracy scores were heavily skewed, we employed transformations to approximate normal distributions for the analyses using conventional performance scores. Latency of correct trials was scored as 1/RT which corresponds with a speed metric (responses per second), whereas accuracy was probit-transformed.

### 2.3. Figural Updating Task

**Figure 2:**
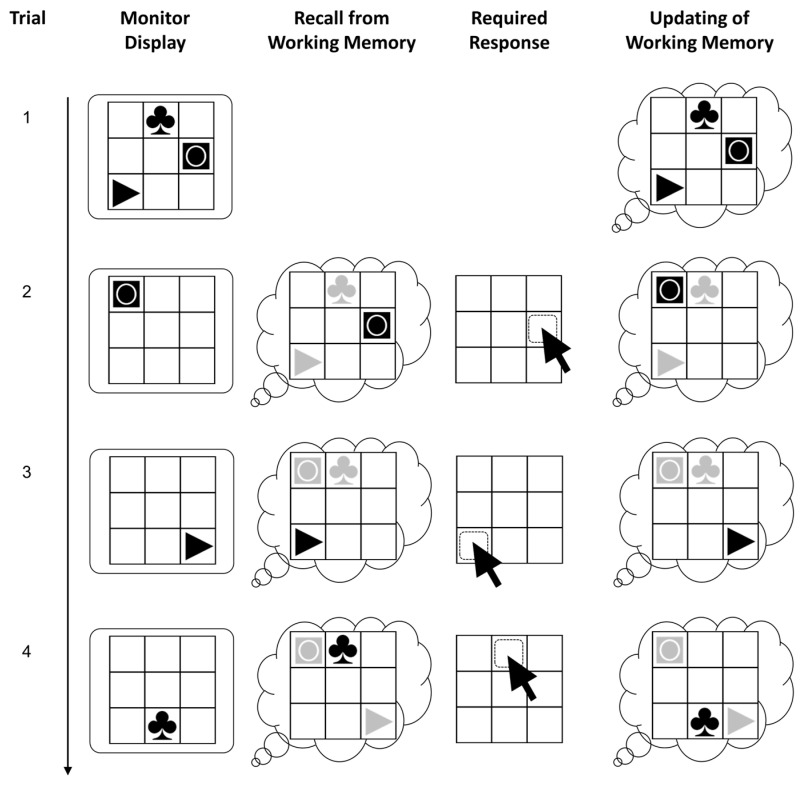
Schematic of the figural updating task. The trials shown here present the first four trials of a block. The monitor display is shown in the leftmost column. The next columns present the recall, response, and updating requirements in the respective trials. Trials 2–4 exemplify 1-back, 2-back, and 3-back recall requirements. In reality, the sequence of recall requirements was pseudo-random and unpredictable for participants.

The capacity of working memory was assessed using a Recall-N-Back paradigm with figural stimuli (see Figure 2) that has been used in previous research (e.g., Schmitz and Wilhelm 2016; Wilhelm et al. 2013). The task requires to update stimulus-location bindings across blocks of trials. Each block started with the presentation of a 3 × 3 grid with one to four simple figures, each in a different cell. After the stimuli disappeared, one figure was shown for the fixed duration of 4000 ms in one of the cells in each trial. Participants were instructed to indicate by mouse-click in which of the cells the currently shown figure had been presented the last time. Then they had to update the new position of that stimulus so they could recall it when shown the next time. Responses were possible only during the fixed presentation interval; however, its sufficiently long duration did not require particularly speedy responding. After giving a response, the stimulus was removed and only the grid was visible for an inter-stimulus interval of 500 ms.

A pseudo-random trial list was used with three practice blocks and 12 test blocks. The blocks had been constructed following a matrix design with different combinations of load (one to four stimuli that had to be remembered) and updating requirements (6, 9, 12 updates). Data from the test blocks were analyzed, excluding load-one blocks, as they do not require working memory. Partial credit scoring (Conway et al. 2005) was applied to compute the scores (i.e., the sum of all correct individual responses).

### 2.4. Decomposing Task-Switching Costs with the Diffusion Model

Parameters of the diffusion model were estimated using HDDM (Wiecki et al. 2013), Version 0.9. The program uses hierarchical Bayesian parameter estimation which has been shown to recover individual differences even for moderate trial numbers adequately in simulation studies (Lerche et al. 2017; Ratcliff and Childers 2015). A mixture model with five percent contamination was specified, and 10,000 posterior samples with 1000 burn-in were drawn (Wiecki et al. 2013). Convergence was checked by inspecting the posterior traces and the Gelman-Rubin $\hat{R}$ statistic.

As parameter estimates were intended for individual differences analyses, a parsimonious model was desired. That is, unnecessary complexity was avoided that could deteriorate the reliability of the estimates. The three core parameters were estimated, namely response caution, drift rate, and non-decision time. However, intertrial variability was fixed to zero. Further, some equality constraints were made in line with earlier findings. Specifically, response caution was set equal across all trial types of a block (as done by Madden et al. 2009; Ong et al. 2019) because the nature of the upcoming trial could not be predicted in the current task. Non-decision time was allowed to vary only between task-switch and repeat trials, but not between response congruent and incongruent trials. This was motivated by multiple-components models of task switching that predict an increase in preparation time to be confined to task switches.

The adequacy of these constraints was checked by means of Bayesian hypothesis tests of the posteriors as well as posterior predictive checks of alternative models that differ in complexity (i.e., the number of estimated parameters). For each of these models, parameters were used to simulate observed RT data (with 500 replicates) to check if their respective distributions closely resembled that of the observed RT data.

### 2.5. Latent Change Score Modeling

Task switching and response congruency effects were estimated by means of latent difference scores (LDS) modeling which avoids some of the limitations of observed difference scores (McArdle and Hamagami 2001; McArdle 2009; Raykov 1999). LDS modeling allows estimating both the difference between experimental conditions on the level of reliable factors as well as its relations with relevant criterion variables. All analyses were conducted with R (R Core Team 2022). The lavaan (Rosseel 2012) and semTools (Jorgensen et al. 2022) packages were used to estimate the structural equation models.

As there was just one indicator task of set shifting in this study, we estimated parameters for each of the three blocks as in Benedek et al. (2014). Technically, the latent difference score (LDS) model assumes strict measurement invariance (equal loadings, intercepts, and residuals) for the conditions that constitute the difference. In the present case, it means that parameters derived from the same block have equal measurement characteristics. However, they could differ across blocks (e.g., as a function of learning or loss of attention). Corresponding residual variances were allowed to covary for this reason.

As a baseline condition, we used response–congruent task-repeat trials. The increased processing requirements of a task switch were modeled using task-switch trials with congruent response mappings to derive a pure estimate of the task-switching effect. Analogously, effects of response incongruency were modeled by using response–incongruent trials from task-repeat trials to estimate the pure effect of response incongruency. The latent mean difference and the relations of the baseline condition and the reliable increment with WMC were tested simultaneously for each diffusion model parameter and performance score. As there was only one indicator task also for WMC, three parcels were built with comparable requirements in terms of load and updating, so that the indicators would be parallel in terms of difficulty and discrimination. Parceling was chosen to derive a parsimonious measurement model (given the only moderate sample size) and because we were not interested in the measurement of visuospatial WMC itself but in its latent relations (see Little et al. 2013).

## 3. Results

### 3.1. Preliminary Analyses of Observed Performance Scores

First, we checked whether the task switching paradigm yielded the expected effects in response times and errors. Descriptive statistics for these conventional performance metrics are given in Table 1, split by trial sequence (task repeat vs. switch), response congruency, and block. Typical effects of task switching and response incongruency were observed across all blocks. The global scores were computed using trials from all conditions (e.g., all switch minus all repeat trials), whereas the specific scores were computed using trials only from the condition where a specific requirement was selectively increased (e.g., congruent–switch minus congruent–repeat trials).

Mean differences were tested with three-way ANOVAs with Block (3), Switch (2), and Incongruency (2) as factors. For the (normalized) RT data, this yielded a main effect of Block (*F* (2, 200) = 75.03, *p* = .001, $\eta_{p}^{2}$ = .43), of Switch (*F* (1, 100) = 451.71, *p* < .001, $\eta_{p}^{2}$ = .82), and of Incongruency (*F* (1, 100) = 129.25, *p* < .001, $\eta_{p}^{2}$ = .56). Further the interaction terms of Block × Incongruency (*F* (2, 200) = 14.39, *p* < .001, $\eta_{p}^{2}$ = .13), and the three-way interaction (*F* (2, 200) = 7.22, *p* = .001, $\eta_{p}^{2}$ = .07) were significant. The latter was driven by the finding that there was a reduced incongruency effect in particular for task repeat trials in the first block in relative terms. Neither the interaction of Block × Switch (*F* (2, 200) = 1.24, *p* = .29, $\eta_{p}^{2}$ = .01) nor the one of Switch × Incongruency were significant (*F* (1, 100) = 1.15, *p* = .29, $\eta_{p}^{2}$ = .01).

An analogous ANOVA for the (normalized) error data yielded a main effect of Switch (*F* (1, 100) = 127.29, *p* = .000, $\eta_{p}^{2}$ = .56) and of Incongruency (*F* (1, 100) = 373.01, *p* = < .001, $\eta_{p}^{2}$ = .79), an interaction of Block × Switch (*F* (2, 200) = 4.38, *p* = .014, $\eta_{p}^{2}$ = .04), and of Block × Incongruency (*F* (2, 200) = 9.75, *p* < .001, $\eta_{p}^{2}$ = .09). There was no significant main effect of block (*F* (2, 200) = 2.21, *p* = .112, $\eta_{p}^{2}$ = .02), no interaction of Switch × Incongruency (*F* (1, 100) = 1.36, *p* = .247, $\eta_{p}^{2}$ = .01), and no three-way interaction (*F* (2, 200) = 1.06, *p* = .348, $\eta_{p}^{2}$ = .01). Overall, these analyses confirmed the effects of task-switching and of response congruency in latencies and errors. However, switching and incongruency effects appeared independently from each other. And they decreased slightly in magnitude across blocks.

### 3.2. Component Processes of Task Switching

**Figure A1:**
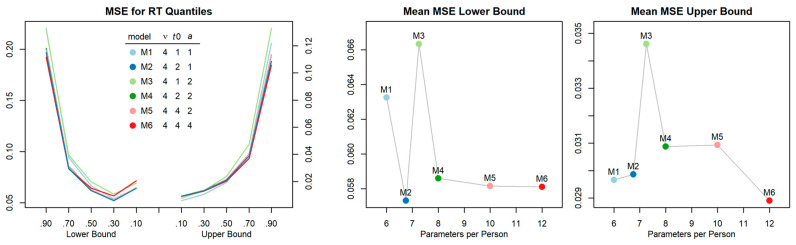
Mean squared error (MSE) for six alternative models fitted to the RT data of the switching task. The numbers in the legend correspond with the conditions the respective parameter was allowed to depend on, i.e., 1 = equal for all conditions, 2 = split by task-switch vs. repeat, and 4 = separate for all four conditions (task-switch × response–congruency).

First, we checked that parameter constraints were tenable, that is, non-decision time was dependent only on task switch vs. repeat and response caution could be set equal across all four conditions. Posterior predictive checks were performed for a series of alternative models with increasing complexity (i.e., independently estimated parameters) and compared in terms of mean squared error (MSE; see Figure A1). The parsimonious model (M2) with response caution (*a*) constrained to be equal across trials of one block, with non-decision time (*t*_0_) depending on task switch vs. repeat, and with drift rate (ν) depending on the combination of task-switch and response congruency was favored by a comparatively low MSE index. By contrast, models assuming identical non-decision times in task-switch and repeat trials (M1, M3) revealed poor fit. At the same time, further increasing the number of parameters (M4–M6) did not improve fit but rather resulted in reproducibility problems.

**Figure A2:**
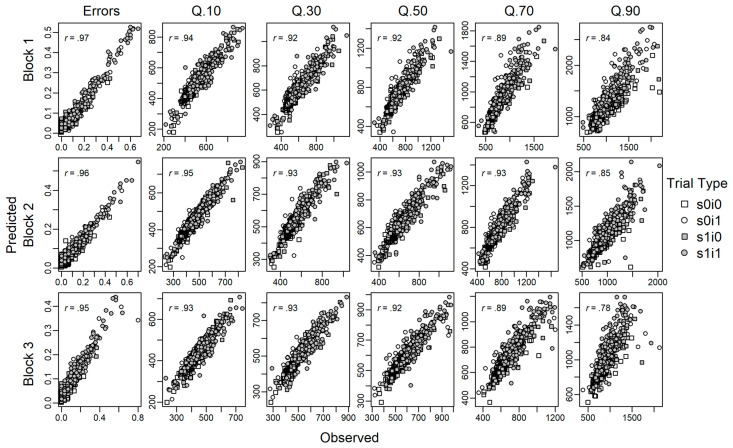
Scatterplot of observed and model-predicted proportion of errors and quantiles of correct responses (in milliseconds) for participants in the respective trial types, as defined by task repeat vs. task-switch (s0; s1) trials with response–congruent vs. incongruent (i0; i1) stimulus attributes.

The specified model (M2) converged well. A graphical inspection suggested the traces of posteriors were stationary. As a more formal test, we also inspected the Gelman-Rubin $\hat{R}$ convergence statistics for all estimated parameters (i.e., all person × condition combinations) which were very close to the desirable value of 1 (*M* = 1.00020, *SD* = 0.00062, range: 0.99989–1.02049). Bayesian hypotheses tests further confirmed that the non-decision time estimates could be equated across response compatibility. This was, in particular, the case for task-repeat trials (probability of difference: *p* = .04, .27, .15 for blocks 1–3), whereas evidence in task-switch trials was more indifferent (probability of difference: *p* = .68, .56, .47, for blocks 1–3). Finally, the posterior predictive checks indicate that the estimated model parameters could decently recover individual differences in the characteristics of the distributions of observed response times (see Figure A2).

**Figure 3:**
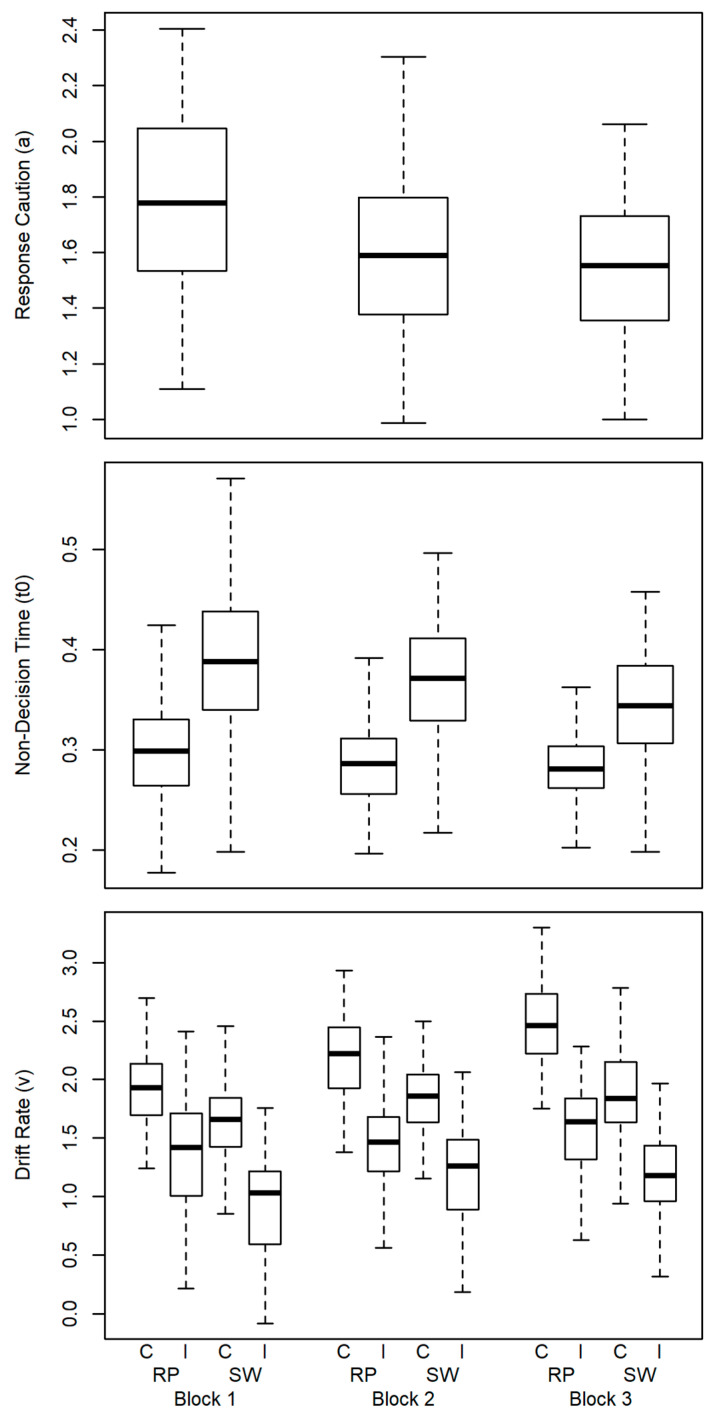
Boxplots of diffusion model (DM) parameters. Upper panel: response caution split by block; middle panel: non-decision time, split by block and task repeat (RP)/switch (SW) trials; lower panel: drift rates, split by block, task repeat (RP)/switch (SW) and response congruent (C)/incongruent (I) trials.

Findings for the mean effects of the diffusion model parameters are depicted in Figure 3. Response caution decreased across blocks, which was confirmed by a one-way ANOVA with Block (3) as the factor (*F* (2, 200) = 64.93, *p* < .001, $\eta_{p}^{2}$ = .39).

Effects in non-decision time were tested using a two-way ANOVA with Block (3) and Switch (2) as factors. This yielded a main effect of Block (*F* (2, 200) = 24.71, *p* < .001, $\eta_{p}^{2}$ = .20) and of Switch (*F* (1, 100) = 273.92, *p* = < .001, $\eta_{p}^{2}$ = .73) and an interaction of both factors (*F* (2, 200) = 18.60, *p* = < .001, $\eta_{p}^{2}$ = .16). The pattern implies that longer non-decision time was observed in task-switch trials relative to task-repeat trials in all blocks. Across blocks non-decision time decreased, in particular, in the task-switch trials.

Effects in drift rate were submitted to a three-way ANOVA with Block (3), Switch (2), and Incongruency (2) as factors. This yielded a main effect of Block (*F* (2, 200) = 107.89, *p* < .001, $\eta_{p}^{2}$ = .52), of Switch (*F* (1, 100) = 411.85, *p* < .001, $\eta_{p}^{2}$ = .81), and of Incongruency (*F* (1, 100) = 750.44, *p* < .001, $\eta_{p}^{2}$ = .88). Further, there was an interaction of Block × Switch (*F* (2, 200) = 17.64, *p* < .001, $\eta_{p}^{2}$ = .15) and Block × Incongruency (*F* (2, 200) = 8.39, *p* < .001, $\eta_{p}^{2}$ = .08), as well as the three-way interaction (*F* (2, 200) = 25.82, *p* < .001, $\eta_{p}^{2}$ = .21). The latter appeared to indicate that drift rates in congruent task-repeat trials were somewhat steeper in the third block. The effects in drift rate indicate largely independent effects of switching and incongruency.

### 3.3. Relations with Visuospatial WMC

**Figure 4:**
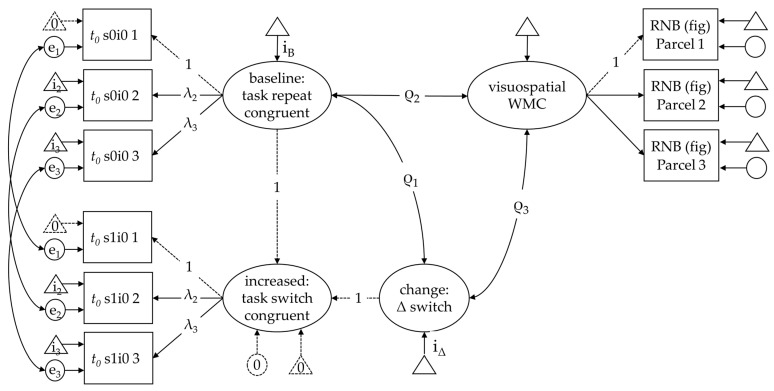
Specification of the latent difference score (LDS) model, as exemplified for the task-switch effect in the diffusion model (DM) non-decision time parameter (*t*_0_), and its relations with visuospatial working memory capacity (WMC) that was assessed using three parallel parcels of the figural Recall-N-Back (RNB) task. Analogous models were estimated for the other parameters. Indicators of the LDS models are systematically named taking into consideration their task switch (s1; s0) and response incongruency (i1; i0) requirements (see Figure 1 for details). Parameters numerically fixed at either 1 or 0 zero are displayed using dashed lines; parameters constrained to be equal are named identically in this figure. The latent correlations between factors (ρ_1_–ρ_3_) are provided in Table 3.

The differential relations with WMC were tested with three latent difference score (LDS) models. One for the task-switch effect in non-decision time, the task-switch effect in drift rates, and the response incongruency effect in drift rates, respectively. All measurement models were identified with substantial loadings (Table A1), the fit was acceptable to good for all models (Table 2) in spite of applying strict invariance constraints in the measurement models (see Figure 4 for details).

First, we tested the relations of WMC with the non-decision time increment that was supposed to reflect task-set preparation (H1). The mean estimates suggested that non-decision time contributed on average with 298 ms to the overall RT in baseline trials (i.e., the non-switch condition), whereas the increment (i.e., the mean difference to the task-switch condition) was 88 ms, where the latter may correspond with the additional time consumed for task-set preparation. Importantly, WMC was not related with individual differences in non-decision time in the baseline condition ($\rho_{2}$ = −.01, *p* = .95), but it was inversely related with the task-switch related increase ($\rho_{2}$ = −.40, *p* < .001) which implies reduced task-switch costs in *t*_0_ for people high in WMC (see Table 3 for details). We checked whether the four persons who did not report being university students affected the results (either by artificially increasing variance or by distorting the pattern of covariation). It turned out that fit, measurement models, and parameter estimates were highly comparable. If at all, the latent relations tended to be larger (WMC–witch *t*_0_: ρ = −.47, *p* < 001.; WMC–Switch v: ρ = .37, *p* = .033; WMC–Incon v: ρ = −.38, *p* = .012).

Next, we tested differences in drift rate (H2) that was assumed to be affected by task-set inertia and other factors that contribute to the state of task readiness. Specifically, the decrease in drift rate and its relations with WMC in case of task-switch trials (reflecting switching costs by inertia; H2a) and in case of incongruent trials (reflecting interference; H2b). Both mean effects were confirmed (Table 3). There was only a trend of a positive relation of WMC with the drift-rates change factor ($\rho_{3}$ = .30, *p* = .09), which implies that people high in WMC tended to have steeper drift rates in task-switch trials in relative terms. Unexpectedly, though, WMC was descriptively negatively related with the drift rate change factor in incongruent trials ($\rho_{3}$ = −.27, *p* = .07), which implies that the decrease in drift rate was even more pronounced for people high in WMC.

To double check that these effects are not pure artifacts of trade-offs in parameter estimation, we also estimated analogous LDS models for the observed performance scores. This replicated conceptually for RT that high WMC is negatively related with task-switching costs (ρ = −.45, *p* < .001) at the expense of an increased interference effects (ρ = .43, *p* = .042). Likewise, effects for classification errors had the same direction but were weaker and remained non-significant. Details are provided in the Appendix A (see Table A2 for the model fit, Table A3 for the latent relations, and Table A4 for the measurement models).

Finally, we tested the relation of WMC with response caution (H3). As individual differences in the estimates of this parameter were correlated across blocks, we modeled response caution as a latent factor across these indicators (with substantial loadings λ = .79, .80, .98, for blocks 1-3; and decent fit: *χ*^2^(8, 101) = 1.96, *p* = .98; RMSEA = .00 [.00−.00]; SRMR = .03; CFI = 1.00; TLI = 1.05). The WMC and response–caution factors were moderately negatively related (ρ = −.28, *p* = .04) which implies that people high in WMC responded less cautiously.

## 4. Discussion

### 4.1. Observed Performance Scores

Effects of task-switching and response incongruency were reflected in both conventional performance metrics. Apparently, task-switch costs in this paradigm with geometric forms were larger in response times, whereas incongruency effects were larger in the errors. However, the effects of switching and incongruency were found to be largely independent of each other in both observed performance metrics.

Task-switch costs in RT decreased slightly across blocks, whereas error effects did not change consistently, suggesting some sort of training that affects, in particular, the consumed time. Generally, these findings replicate previous research that participants display reduced task-switch cost with practice (Minear and Shah 2008; Zhao et al. 2020). Interestingly, incongruency effects in RT rather increased across blocks, while errors decreased tendentially only from the first to the later blocks. This pattern of effects suggest that people may become less cautious with task experience. Diffusion modeling may shed light at which factors contribute to the observed task-switch cost, the incongruency effects, and the changes across blocks.

### 4.2. Process-Components of Task Switching

As suspected, response caution decreased across task blocks, suggesting that participants became more confident with practice. Further, and replicating earlier findings (Karayanidis et al. 2009, 2010; Schmitz and Voss 2012, 2014), we found non-decision time to be increased in task-switch trials as compared with task-repeat trials. In line with additional process accounts of task switching (De Jong 2000; Rogers and Monsell 1995; Rubinstein et al. 2001), this increase was interpreted as reflecting switch-related preparatory processes. Across blocks, there was a slight reduction of the difference between switch and repeat trials in non-decision time. This suggest that less time is consumed for preparation with practice. The exact nature of preparatory processes is unresolved, but could likely correspond with accounts on re-programming of the cognitive system (Ruthruff et al. 2001) or top-down biasing of the relevant task-demand units (Gilbert and Shallice 2002). The latter may be described as well as loading the task set into working memory (Goschke 2000; Mayr and Kliegl 2000) or loading it into the region of direct access (Risse and Oberauer 2010).

Drift rates were affected by switching and incongruency, but both effects were apparently independent of each other. Generally, the pattern in drift rates plausibly reflects task-set inertia (Allport et al. 1994; Allport and Wylie 2000). Inertia effects can be both beneficial as in task-repeat trials and detrimental as in task-switch trials. The difference between both conditions likely comprises both effects. Further, inertia may exist in terms of persisting activation of inhibition (Schuch and Koch 2003) which could be described as differential levels of task readiness on a dimensional scale. Generally, the mean-effects for the diffusion model parameters can be reconciled with multiple component models of task switching (Mayr and Kliegl 2003; Ruthruff et al. 2001), thereby replicating previous research (Boag et al. 2021; Ging-Jehli and Ratcliff 2020; Karayanidis et al. 2009, 2010; Klauer et al. 2007; Madden et al. 2009; Miller et al. 2018; Schmitz and Voss 2012).

### 4.3. Relations with Visuospatial WMC

Going beyond the replication of mean effects in diffusion model parameters, the current study aimed at testing their relations with visuospatial WMC. Consider first the relation of WMC with the task switch effect in non-decision time that was interpreted as reflecting task-set preparation. Specifically, we tested if the non-decision time increase in task-switches would be less pronounced for people high in WMC (H1). This was confirmed, revealing (inverse) relations with the non-decision time effect in about the magnitude as those previously observed for the relations between WMC and shifting (Feng et al. 2022; Himi et al. 2019; Markett 2013; Oberauer et al. 2003). The inverse relation with non-decision time costs can be reconciled with the notion that the capacity for updating bindings constitutes WMC (Oberauer et al. 2007; Wilhelm et al. 2013), and likewise it would facilitate the updating of the S-R bindings that constitute a task set. Further, it appears straight-forward that people with higher WMC can represent the individual S-R mapping rules more completely in working memory, which should reduce the efforts to reactivate them again in case of a task switch. Indeed, the look-up table effect (Dreisbach et al. 2006, 2007) suggests that switch-costs disappear when people represent mapping rules simultaneously, so that switching becomes obsolete.

The finding that non-decision-time related task-switch costs are inversely related with WMC can be reconciled with the notion that they are particularly indicative of cognitive functioning. At least, it fits in well that they maturate comparatively late in adolescence (Weeda et al. 2014) and decline again with age (Ging-Jehli and Ratcliff 2020; Karayanidis et al. 2011; Whitson et al. 2012), in particular in case of cognitive impairments (Velichkovsky et al. 2020). Given that non-decision time decreased particularly in task-switch trials (but less so in task-repeat trials) suggests that task experience reduces the time required or devoted to preparatory processes. This may contribute to the reduced switch costs previously observed in the conventional RT metric (Minear and Shah 2008; Zhao et al. 2020). The finding of reduced preparation times with practice can be reconciled as well with the previous finding that training effects are most pronounced when the cue-stimulus interval is short; hence, when there is only limited time for preparation (Zhao et al. 2020).

It needs to be discussed, though, that the duration of these proactive control processes may reflect both, the time required or the time devoted to set preparation. In fact proactive control may be exerted as means of compensation to reduce effects of interference (Whitson et al. 2014), and this may be particularly relevant for people who may suffer from increased interference, like the elderly. Longer preparation could either reflect a consciously applied strategy or result from largely unaware conditioning as reinforced by the success and failure of previous classifications. Hence, the inverse relation of WMC with non-decision time in task-switch trials could either indicate that high-WMC participants needed less time for task-set preparation or that they invested less time into preparation, possibly resulting from higher confidence. The tendentially increased interference effect in terms of drift-rates (see below) suggests the latter interpretation could hold as well. Interestingly, it has been shown for an experimental paradigm that requires task switching, that adolescents display a minimum in non-decision time when between 14 and 16 years old (von Krause et al. 2022), which parallels a critical developmental phase characterized by high levels of impulsivity and sensation-seeking (Harden and Tucker-Drob 2011). Generally, it has been discussed already that preparatory processes prior to stimulus onset deserve considerably more attention (Teichert et al. 2016).

Consider next the relations of WMC with the drift rate effects of task switching (H2a) and response–congruency (H2b). Both effects, that is, decreases in drift rates in the more complex condition, missed conventional criteria of significance in the present study (.05 < *p* < .10). However, the task-switch effect tended to point in the predicted direction. Specifically, high-WMC participants had a less pronounced decrease in drift rates in task-switch trials relative to task-repeat trials. This seems plausible, as more capable persons have been shown to possess steeper drift rates in the specific requirements of more complex elementary cognitive tasks (Goecke et al. 2021; Lerche et al. 2020), although the latter tasks did not require task-switching.

Contrary to expectations, visuospatial WMC tended to be inversely related with the incongruency change score. This suggests that high-WMC participants had somewhat stronger decreases in drift rates, indicating that they suffered more from proactive interference. Interestingly, this effect was statistically significant also in the conventional RT scores. Thus, it does not appear to be a pure artifact of the parameter estimation. The increased interference of high ability participants could reflect the adverse flip side of high WMC: The more complete participants can represent all mapping rules in memory, the more likely currently irrelevant rules could interfere at response selection. Alternatively, the increased interference could result from insufficient task-set preparation that could also account for the previously discussed shorter non-decision time effects of high-WMC participants in this sample. The finding that high-WMC participants suffer more from interference is at odds with WMC-accounts on attentional resources (Engle 2002; Kane et al. 2004). Given that the latter have received some support in the literature, the non-predicted (tendential) effect observed here should be considered with caution and replication with more diverse samples and with different indicator tasks is desirable.

Consider now the relation of WMC with response caution (H3) which could be tested as a secondary hypothesis using the current data. WMC was found to be moderately negatively related with response caution, confirming previous findings with data from elementary cognitive tasks (Schmitz and Wilhelm 2016; Schmitz et al. 2018). One possible explanation is that more capable persons tend to take risks due to a higher ability self-concept (Freund and Kasten 2012). However, a more likely explanation may be that more capable persons pay less caution only when the task is easy. This does not rule out that they are more cautious when the task is truly difficult. Such an adaptive strategy of response caution has been demonstrated for diverse ability tests (Becker et al. 2016; Goldhammer et al. 2015). From an applied perspective, the finding that response caution varies differentially calls for caution and suggests modeling this potential trade-off to avoid a bias in the performance metric (see Liesefeld and Janczyk 2019, for an overview and discussion).

It was surprising that visuospatial WMC did not reveal any meaningful relation with parameters of the baseline condition, that is, response–congruent task-repeat trials. However, the absence of a relation with baseline non-decision time could be expected given that this parameter should largely reflect motor time (Ratcliff and Rouder 1998; Voss et al. 2004). The only moderate and statistically non-significant positive relation with drift rate was weaker than expected. Previous research has demonstrated more substantial relations with drift rates modeled from ECTs (Schmiedek et al. 2007; Schmitz and Wilhelm 2016) and also for the conventional response–time scores (Deary 2001; Sheppard and Vernon 2008). Possibly, the baseline condition in this study was too simple and the highly overlearned classifications (e.g., blue → right) had limited requirements on WMC.

### 4.4. Implications for Ability Research and Assessment

Task-switching paradigms could potentially complement ability assessment as a measure of flexibility. However, these paradigms have strengths and limitations. They are much simpler (Beckmann et al. 2017; Birney and Beckmann 2022) than other tasks used for the assessment of cognitive flexibility such as complex problem solving scenarios (Beckmann et al. 2017; Dörner and Funke 2017) or experiential learning tasks (e.g., the IGT, Bechara et al. 1994, or the WCST, Berg 1948). Therefore, one advantage of task-switching paradigms is that their requirements and component processes are much better understood (Koch et al. 2018). High specificity in task requirements implies that these task cannot grasp the entire complexity of flexible operations (Yu et al. 2019) and key-features such as the originality of responses (Carroll 1993).

Generally, more research is required to understand the validity of task-switching paradigms in the nomological network of established ability constructs. Some research has confirmed that task switching is moderately related with working memory capacity (Feng et al. 2022; Himi et al. 2019; Markett 2013; Miyake et al. 2000; Oberauer et al. 2003). It needs to be discussed, though, that task switching requires several component processes (Mayr and Kliegl 2003; Ruthruff et al. 2001), including task-set preparation (De Jong 2000; Rogers and Monsell 1995; Rubinstein et al. 2001) and coping with task-set inertia (Allport et al. 1994; Allport and Wylie 2000). The estimates for the component processes appear to be sufficiently reliable, as can be inferred from the factorial saturation of the indicators (i.e., their high factor loadings). The validity of the markers of component processes needs to be demonstrated more thoroughly in future research. The present study suggests that task-set preparation is related with WMC. At least, this was shown using one paradigm each with figural stimuli. However, it is unresolved which processes contribute to the effect in non-decision time, namely, to what extent does it reflect efficiency of preparation or investment into preparation as a means of proactive control (Whitson et al. 2014). Effects in drift-rate were inconsistently related with WMC. Shifting performance tended to be positively related, and interference control was negatively related. This non-predicted and statistically non-significant pattern should be interpreted with caution and replication is required. One could speculate on grounds of inertia theory (Allport and Wylie 2000) and backward inhibition (Schuch and Koch 2003) that drift-rate effects are related with inhibition. The latter could potentially also contribute to the moderate relations of (observed) task-shifting and inhibition scores (Miyake et al. 2000; Miyake and Friedman 2012). However, speed of processing should be considered as a major confounding variable (Wilhelm et al. 2013); in particular, when drift-rate effects are used as indicators. In any case, more research is required to understand possible relations of the component scores of task-shifting with established ability indicators.

### 4.5. Limitations and Future Research

This study offers preliminary evidence that separable component processes of task-shifting are differentially related with working memory capacity, as shown for the visuospatial domain. However, the correlational design does not allow testing of the direction of causality. It appears plausible that individual differences in the functional capacity to update bindings facilitates task-set preparation. However, it could be argued that shifting is also required in WMC paradigms that are typically complex and necessitate shifting attention between specific processes. This is also one of the reasons why we did not use complex span tasks in this study that require shifting between primary and secondary tasks.

A major limitation is the use of only one task for each construct. Future research would profit from multiple indicator tasks to reduce issues of task-specificity and process impurity (Miyake et al. 2000; Stahl et al. 2014). Further, more broadly defined constructs could be more substantially related with other ability constructs or real-life variables. However, this is an empirical question since paradigms that are assumed to assess executive control tend to be only moderately related. At least, the similarity of processing requirements appears to affect the magnitude of relations between experimental paradigms (Himi et al. 2019; Miyake and Friedman 2012), and relations between specific indicator tasks have been shown to vanish when speed of processing is controlled for (Wilhelm et al. 2013). This has challenged the view that executive control is centrally organized at all; instead, distributed control systems have been proposed as an alternative account (Zink et al. 2021). This implies that the results observed here for one particular task-switching paradigm with geometric shapes and one particular figural updating task could be confined to the visuospatial domain of working memory. Hence, replication is strongly recommended to determine the generality of the described findings. Future research should use different and more diverse indicator tasks and should follow a generally more multivariate design.

The present study comprised a moderate sample of mostly university students who participated for course credit. It is not unlikely that this could have affected the adjustment of response caution or the investment into task-set preparation. Both component processes have been shown to be reduced in younger people, albeit at different ages, with non-decision time revealing a minimum in youth (14–16 years), whereas response caution in young adulthood (at ca. 20 years). Replication across a broader age range and with people of diverse educational background would be desirable.

Another limitation pertains to the majority of female participants in this study. Naturally, there could be gender differences in cognitive flexibility. However, previous research suggests that there are neither sex differences in task-switching nor in dual-tasking while controlling for working memory, processing speed, spatial abilities, and fluid intelligence (Hirsch et al. 2019). Likewise, another study using a large sample (N = 5271, Reimers and Maylor 2005) did not find a gender difference in local task-switch costs, but females revealed larger mixing costs (non-switch trials in mixed-task blocks vs. task-pure blocks) in response times. This pattern suggests that gender does not affect task-switch specific mechanisms, but it may affect global differences in block defaults, such as the setting of response caution. Another study using fMRI confirmed the absence of gender differences in the behavioral measures, while males revealed stronger neural activation in relevant brain regions (prefrontal, left parietal, and the right insula, Kuptsova et al. 2015) which was interpreted by the authors as indicative of additional compensation in males. In summary, previous evidence has not found substantial gender differences in observed task-switch costs. Gender differences in cognitive flexibility, thus, appear to be more of a stereotype than reality (Hirsch et al. 2019). However, gender differences in component processes have not been studied in previous research, and should be a research objective in future studies.

In the present analyses, we modeled factors for diffusion-model parameters. This has been applied successfully in previous research to cope with parameter estimation errors (Schmiedek et al. 2007; Schulz-Zhecheva et al. 2016). Latent-variable modeling can efficiently reduce potential bias when errors in the indicators are independent. Given that parameters were estimated hierarchically in this study, the independence assumption may be likely violated.

## 5. Conclusions

Task-switching is a comparatively well-understood paradigm that may be used for the assessment of cognitive flexibility. In turn, task-switching has been theorized to comprise component processes such as task-preparation and task execution, where the latter can be affected by task-set inertia. Replicating previous research, we sought to decompose these processes by means of diffusion modeling. Preparatory processes appear to consume (more) time in task-switches as compared to task-repetitions. However, these effects decrease with practice across blocks. Further, adverse effects of task-switches and response incongruency decreased processing efficiency during task execution. Differential correlations with working memory capacity suggest that high WMC was associated with shorter preparation time in the current sample. This could likely reflect that high WMC facilitates set-preparation. Alternatively, it may indicate less investment into set preparation as a proactive control mechanism. The relations of WMC with processing efficacy during task execution were inconsistent in the present study and call for replication. Finally, it turned out that a more capable person exercised less response caution.

## Figures and Tables

**Table 1:** Mean (SD) for the observed performance scores.

Trial Type		Response Time (ms)			Errors		
Switch	Incon	Block 1	Block 2	Block 3	Block 1	Block 2	Block 3
0	0	703 (136)	619 (96)	583 (88)	.035 (.066)	.045 (.068)	.033 (.054)
	1	720 (149)	675 (119)	650 (122)	.147 (.127)	.143 (.112)	.129 (.136)
1	0	825 (184)	743 (156)	697 (119)	.056 (.074)	.066 (.073)	.073 (.081)
	1	886 (199)	795 (165)	756 (127)	.238 (.177)	.192 (.154)	.199 (.140)
Switch Effect		145/122	122/124	110/114	.056/.021	.035/.021	.055/.040
Incon Effect		39/17	54/56	62/66	.147/.112	.112/.098	.111/.096

Trial type is defined by the combination of Switch (0 = task repeat, 1 = task switch) and Incon (0 = congruent, 1 = incongruent). The switch and incon effects are given as global effect (averaged across all trials) and a specific effect (only trials with specific requirement included), see text for further information.

**Table 2:** Fit of the LDS models for DM parameters.

Model	*χ*^2^ (*df*, *N*), *p*	RMSEA [95% CI]	SRMR	CFI	TLI
switch *t*_0_	41.32 (28, 101), .05	.07 [.00;.11]	.06	.97	.96
switch ν	32.70 (28, 101), .25	.04 [.00;.09]	.08	.98	.98
incon ν	42.51 (28, 101), .04	.07 [.02;.11]	.10	.96	.94

Latent difference score (LDS) models: switch = model for task-switch effect; incon = model for response incongruency effect. Diffusion model (DM) parameters: *t*_0_ = non-decision time, ν = drift rate.

**Table 3:** Parameter Estimates of LDS models for DM parameters.

	Mean Baseline		Mean Change		Correlation Baseline-Change		Correlation WMC-Baseline		Correlation WMC-Change	
Model	i_B_	*p*	i_Δ_	*p*	ρ_1_	*p*	ρ_2_	*p*	ρ_3_	*p*
switch t_0_	.298	.000	.088	.000	.151	.211	−.011	.933	−.405	.000
switch ν	1.938	.000	−.293	.000	−.324	.027	.161	.214	.301	.082
incon ν	1.945	.000	−.619	.000	.048	.754	.193	.136	−.269	.073

Models: switch = model for task-switch effect; incon = model for response incongruency effect. Diffusion model parameters: *t*_0_ = non-decision time, ν = drift rate. Latent means: i_B_ = mean of the baseline factor; i_Δ_ = mean of the change factor. Latent Correlations (ρ_1_–ρ_3_) denote standardized relations between factors.

## Data Availability

Data and analysis scripts are provided in an online repository (OSF): https://osf.io/6rczd/?view_only=1abc602a16e849bca3748a2c4deebe8b.

## Appendix A

**Table A1:** Factor loadings of LDS measurement models for DM parameters.

	Baseline Factor			Increased Factor			WMC Factor		
Model	λ_11_	λ_21_	λ_31_	λ_12_	λ_22_	λ_32_	λ_13_	λ_23_	λ_33_
switch *t*_0_	.741	.867	.679	.863	.938	.820	.691	.413	.733
switch ν	.562	.701	.847	.559	.698	.845	.654	.409	.777
incon ν	.584	.802	.785	.680	.866	.853	.662	.416	.762

λ = fully standardized factor loadings in the latent difference score (LDS) model for the diffusion model (DM) parameters.

**Table A2:** Fit of the LDS models for observed performance scores.

Model	*χ*^2^ (*df*, *N*), *p*	RMSEA [95% CI]	SRMR	CFI	TLI
switch RT (norm)	47.90 (28, 101), .01	.08 [.04;.12]	.08	.97	.96
switch Error (norm)	26.75 (28, 101), .53	.00 [.00;.07]	.06	1.00	1.01
incon RT (norm)	64.82 (28, 101), .00	.11 [.08;.15]	.08	.94	.93
incon Error (norm)	38.31 (28, 101), .09	.06 [.00;.10]	.08	.95	.93

Latent difference score (LDS) models: switch = model for task-switch effect; incon = model for response incongruency effect. The observed scores were transformed to normalize their distribution (high scores indicate poor performance): RT (norm) = 1/RT*(-1); Error (norm) = probit(%Errors).

**Table A3:** Parameter estimates of LDS models for observed performance scores.

	Mean Baseline		Mean Change		Correlation Baseline-Change		Correlation WMC-Baseline		Correlation WMC-Change	
Model	i_B_	*p*	i_Δ_	*p*	ρ_1_	*p*	ρ_2_	*p*	ρ_3_	*p*
switch RT (norm)	−1.618	.000	.253	.000	.200	.118	−.262	.025	−.449	.000
switch Error (norm)	−2.417	.000	.378	.000	−.374	.077	−.084	.536	−.147	.569
incon RT (norm)	−1.634	.000	.070	.000	.250	.233	−.292	.014	.428	.042
incon Error (norm)	−2.384	.000	1.076	.000	−.545	.000	−.068	.600	.199	.241

Models: switch = model for task-switch effect; incon = model for response incongruency effect. The observed scores were transformed to normalize their distribution (see Table A2 note). Latent means: iB = mean of the baseline factor; i_Δ_ = mean of the change factor. Latent correlations (ρ_1_–ρ_3_) denote standardized relations between factors.

**Table A4:** Factor loadings of LDS measurement models for observed performance scores.

	Baseline Factor			Increased Factor			WMC Factor		
Model	λ_11_	λ_21_	λ_31_	λ_12_	λ_22_	λ_32_	λ_13_	λ_23_	λ_33_
switch RT (norm)	.794	.907	.831	.836	.929	.868	.641	.395	.799
switch Error (norm)	.572	.733	.712	.543	.707	.685	.635	.397	.804
incon RT (norm)	.679	.900	.800	.709	.914	.824	.646	.418	.779
incon Error (norm)	.730	.755	.630	.672	.699	.567	.608	.388	.840

λ = fully standardized factor loadings in the latent difference score (LDS) model for the observed performance scores. These were transformed to normalize their distribution (see Table A2 note).
